# Effect of rotary cutting instruments on the resin-tooth interfacial ultra structure: An in vivo study

**DOI:** 10.4317/jced.51362

**Published:** 2014-12-01

**Authors:** Sudhir Sherawat, Sanjay Tewari, Jigyasa Duhan, Alpa Gupta, Rakesh Singla

**Affiliations:** 1 Consultant, Department of Conservative Dentistry & Endodontics, Post Graduate Institute of Dental Sciences, Rohtak; 2 Professor and Head, Department of Conservative Dentistry & Endodontics, Post Graduate Institute of Dental Sciences, Rohtak; 3 Professor, Department of Conservative Dentistry & Endodontics, Post Graduate Institute of Dental Sciences, Rohtak; 4 Post Graduate Demonstrator, Department of Conservative Dentistry & Endodontics, Post Graduate Institute of Dental Sciences, Rohtak; 5 Reader, Department of Conservative Dentistry & Endodontics, JCD dental college, Sirsa, Haryan

## Abstract

Objectives: To evaluate the effect of cutting teeth with different types of burs at various speeds on surface topography of tooth surface and interfacial gap formation at resin-tooth interface. Material and Methods: The human molars were divided into seven groups: Diamond bur in airrotor (DA) & micromotor (DM), crosscut carbide bur in airrotor (CCA) & micromotor (CCM), plain carbide bur in airrotor (CA) & micromotor (CM) and #600-grit silicon carbide paper (SiC). In five samples from each group Class II box-only cavities were restored. The occlusal surface of four teeth per group was flattened. Two out of four teeth were acid etched. Teeth were subjected for scanning electron microscopy (SEM). Results: Interfacial gap was observed in all groups with no significant difference. SEM observations revealed CA, CCA & DA were coarser than CM, CCM, DM and SiC. SEM of etched tooth surfaces revealed complete removal of amorphous smear layer in CA & CM, partial removal in CCA, CCM, DA & DM and no removal in SiC. Conclusions: Selecting an appropriate bur and its speed may not play an important role in bonding in terms of interfacial gap formation. Variable changes were observed in surface topography with different burs before and after acid etching.

** Key words:**Surface topography, resin-tooth interface, interfacial gap, bonding.

## Introduction

Dentin bonding is a complex phenomenon (1,2). High organic content, tubular structure with the presence of odontoblastic processes, continuous moist conditions due to the presence of dentinal fluid, intratubular pressure, permeability and the forma-tion of the smear layer during cavity preparation makes dentin a sensitive substrate for bonding. Hence, the research on bond strength of various adhesive systems to dentin under different conditions is the area of interest for dentists. Most of the in vitro studies were conducted using flat surface of dentin prepared with SiC abrasive paper for dentin bonding However, clinical conditions are entirely different.

Due to the different configuration of flat surfaces in vitro studies and complex cavities prepared in clinical situation, the bonding results may not be same in both situations. Box-like Class I cavities in which the walls have equal dimensions have a configuration (C) factor of 5; whereas a flat surface, as in veneering, would have a C-factor of 1 (3). Laboratory studies conducted at a C factor of 1 tend to overestimate bonding performance compared with complex cavity preparations with high C-factors (3,4).

Surface topography has significant influence on dentin bonding. It directly affects the surface wetting by bonding resins (5). Surface irregularities also affect the quality of bonding by varying the surface area available for adhesion (6,7). Since the abrasive paper used in most of bonding studies in vitro and the rotary cutting instruments used in clinical conditions are likely to produce different surface characteristics, the effect of these instruments and their speed on bonding needs to be evaluated.

Adhesion to dentin is influenced by the characteristics of smear layer created by a rotary cutting instrument. Smear layer reduces dentin permeability; and impedes the contact of adhesive resin with dentin. Rotary instruments create smear layers of varying resistance to acid removal (8); and thus yield different bond strength results.

Several in vitro studies have investigated the effect of dentin surface preparation with different instruments on bond strength and micromorphology at resin-dentin interface. Ayad *et al.* (9) reported that the micro tensile bond strength values were significantly higher with tungsten carbide finishing burs and smooth dentin surfaces for both total etch and self etch adhesives used in the study. Various other studies have also reported higher bond strength for carbide burs than for diamond burs (10-13). Still other researchers have also reported no significant difference in bond strengths with different preparation methods, specifically with total etch adhesive system (14). However, the results of these in vitro studies may not be transferrable to the complex clinical situations as they don’t take into account the pulpal pressure and dentinal fluid which have a significant influence on dentin bonding in clinical situations. Hannig & Friedriehs (15) reported that the internal seal of totally bonded adhesive restorations varies considerably in vivo and in vitro. Significantly better results are obtained when the bonding parameters are tested in vitro than in vivo conditions. To the best of our knowledge, no study till date has been reported to compare the effect of various rotary cutting instruments on the dentin adhesion performed under clinical conditions. Therefore, the present study evaluated the surface topography of cut and acid etched dentin substrate after preparation by different rotary cutting instruments at different speeds and to determine the in vivo efficacy of different burs at different speeds in achieving a gap free internal adaptation between resin restorative material and dentin.

## Material and Methods

The study was approved by the institutional review board of Pandit Bhagwat Dayal Sharma Institute of Health Sciences, Rohtak, Haryana, India. Sixty three caries-free human molars scheduled for extraction were used in the study with the informed consent of the donor. The teeth were divided into the following seven groups, with nine teeth in each group, according to the type of cutting instrument and speed used A) Fine grit straight fissure diamond bur in air rotor (DA). B) Fine grit straight fissure diamond bur in micromotor (DM). C) Crosscut fissure carbide bur in air rotor (CCA). D) Crosscut fissure carbide bur in micromotor (CCM). E) Plain fissure carbide bur in micromotor (CM). F) Plain fissure carbide bur in air rotor (CA). G) #600-grit silicon carbide abrasive paper (SiC).

Five samples from each group were used for evaluation of interfacial gap and four for surface topography evaluation. Class II proximal box-only cavities of standard dimensions 4-mm buccolingual, 4- mm occlusogingival and 2-mm mesiodistal, with facial and lingual walls straight and parallel to each other, were prepared. The standard dimensions included both the enamel and dentin surfaces. In all groups, an initial preparation was done by fine-grit straight-fissure DM, and then the surface was finished with different types of burs ( Table 1). Teeth in the air rotor groups A and C were prepared with burs rotating in a dental turbine at a high speed of 150,000 rpm (Contraangle PANA AIR T air rotor handpiece, NSK, Nakanishi Inc, Tochigi-ken, Japan). Teeth in Groups B, D and E were prepared with their respective burs mounted in a contra angle micromotor handpiece at 40,000 rpm (NSK, Nakanishi Inc). Handpieces were hand-held to simulate clinical conditions. In Group F, #600-grit silicon carbide abrasive paper was used. A small piece of this paper was glued to a metallic blank bur with cyanoacrylate. This blank was mounted in the micromotor at slow speed (40,000 rpm), and 30 passes were made across the tooth surface under copious airwater spray. The surface was then prepared by 10 strokes with the same mounted silicon carbide abrasive paper on a blank bur when the micromotor was not rotating to create uniform scratches. The abrasive paper was changed as soon as it got distorted.

**Table 1:**
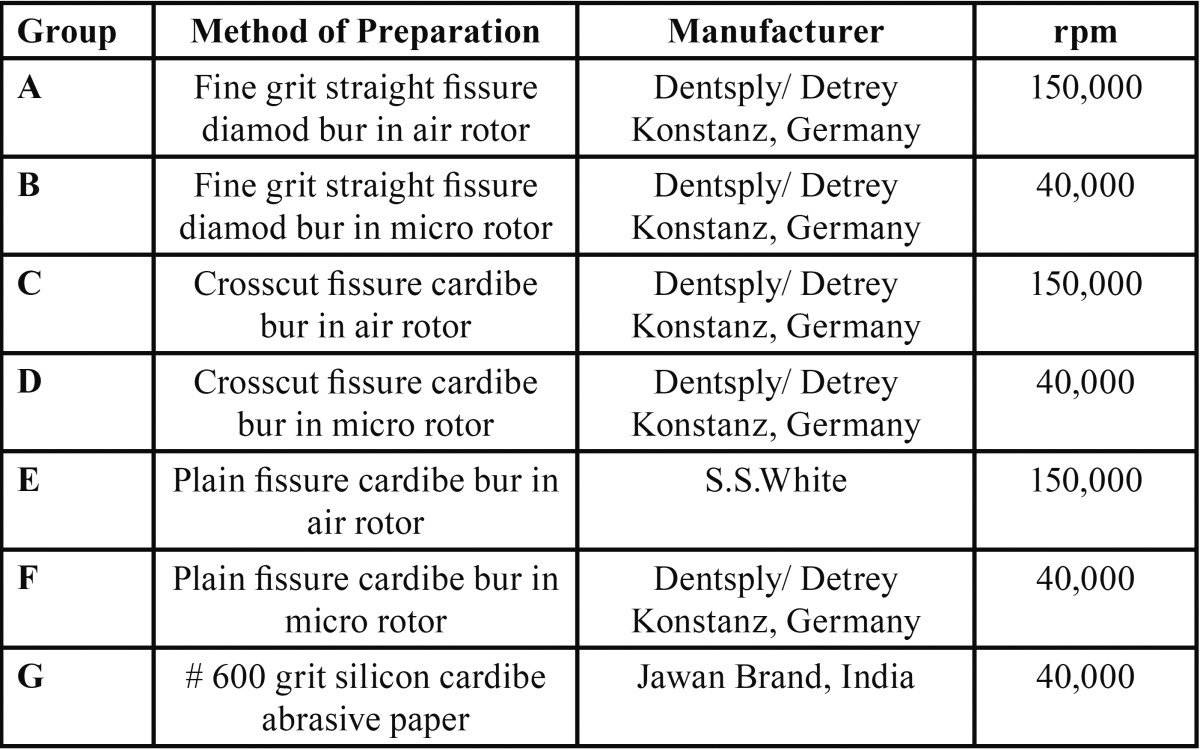
Identification of groups by dentin surface preparation.

In all groups, the tooth surface was prepared by the bur under copious air-water spray until uniform scratches by each bur covered the entire tooth surface. Each bur was changed after preparing three cavities.

The prepared enamel surface was etched with 37% ortho phosphoric acid (DPI tooth conditioning gel, Dental Products of India Ltd, Mumbai, India) for 30 seconds, with the help of a brush. Then, the et chant was applied to the prepared dentin surface for 15 seconds ( Table 2). This resulted in enamel etching for 45 seconds and dentin etching for 15 seconds. The et chant was rinsed with distilled water for 10 seconds with a high force of combined air-water spray and blot dried with a cotton pellet to keep the surface moist. For this purpose, excess water from a cotton pellet saturated with water was removed by blotting it on a gauze pad before using the pellet to blot the tooth. Then, one coat of the bonding agent Prime and Bond NT (Dentsply/DeTrey, Konstanz, Germany) was applied with a bristle brush. The surface was kept wet for 20 seconds and gently air- dried for five seconds to a glossy surface, then photopolymerized using a light intensity of 600 mW/cm2 for 10 seconds. A mylar strip was now applied to cover the proximal box. Spectrum TPH, the hybrid resin composite, (Dentsply/DeTrey) was packed in 2-mm thick horizontal increments. The last layer was made flush with the enamel cavosurface margins. Each layer was exposed to the curing light for at least 40 seconds from the occlusal side. Teeth were extracted immediately after restoration, cleaned and stored in distilled water at 370C for a period not exceeding 4 weeks.

**Table 2:**
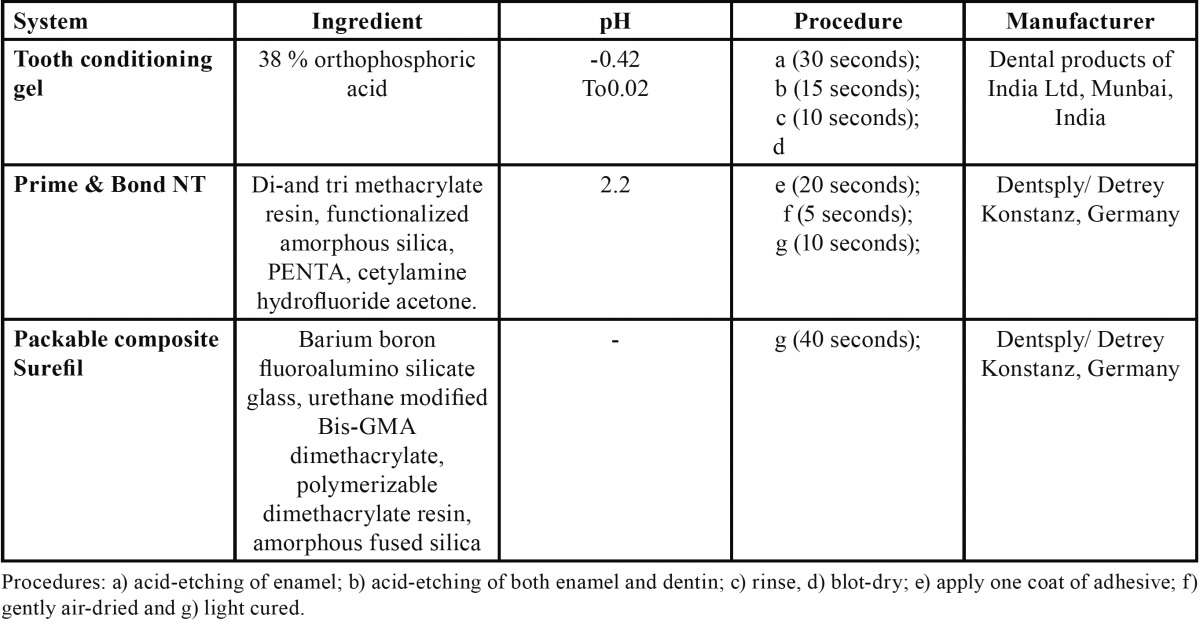
Materials udes for Bonding.

The specimens were sectioned vertically in a mesiodistal plane through the centre of the restoration and polished with Enhance system disks and paste (Dentsply Detrey/ Germany). After fixation in 10% formalin for 24 hours, sections were decalcified in 6N HCl for 30 seconds, rinsed in distilled water, deproteinized by 5 minute immersion in 5.25% NaOCl and rinsed with distilled water. After acid base treatment, specimens were subjected to dehydration in ascending grades of ethanol, mounted on aluminium stubs and further dried in vacuum. Gold sputter coating was carried out under reduced pressure in an inert argon gas atmosphere in Agar Sputter Coater P7340 and the specimens were examined under Scanning Electron Microscope (Leo 435 VP) operated at 15 kV. Photomicrographs of the axial and gingival tooth-resin interface were taken at 500X.

The measurements of interfacial gap were recorded at five predetermined equidistant points, and the values were averaged. The measured interfacial gap values were analyzed using ANOVA/T- test at <0.05 level of significance.

-Specimen preparation for surface topography

The occlusal surface of 28 teeth was cut flattened up to mid-dentin by fine grit straight fissure diamond bur in air rotor. Then the surfaces were prepared with different type of burs at varying speeds. In order to understand the effect of conditioning on the dentinal surfaces prepared by different types of burs, half of the teeth were acid etched with 38% phosphoric acid and the other half were not given any further treatment. Specimens were dehydrated in ascending grades of ethanol, gold coated and examined under scanning electron microscope at 500X magnification.

## Results

**Figure 1:**
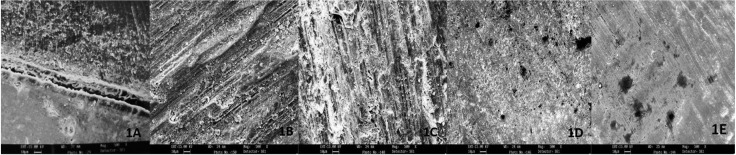
A) SEM view of the interfacial gap at resin-dentin interface at 500x, B) SEM view of tooth surface prepared with fine grit straight fissure diamond bur in air rotor at 500x, C) SEM view of tooth surface prepared with fine grit straight fissure diamond bur in micro motor at 500x, D) SEM view of tooth surface prepared with crosscut fissure carbide bur in air rotor at 500x, E) SEM view of tooth surface prepared with crosscut fissure carbide bur in micro motor at 500x.

The mean interfacial gap (Fig.1A) at axial & gingival tooth-resin interface for each group observed in scanning electron microscope examination is shown in ( Table 3). These results shows that there was no significant difference in all the groups, whether we used Fine grit straight fissure diamond bur, Crosscut or Plain fissure carbide bur and #600-grit silicon carbide abrasive paper in air rotor or micro motor on axial or gingival tooth surface. Less marginal gap was observed at enamel margins as compared to dentin margins.

**Table 3:**
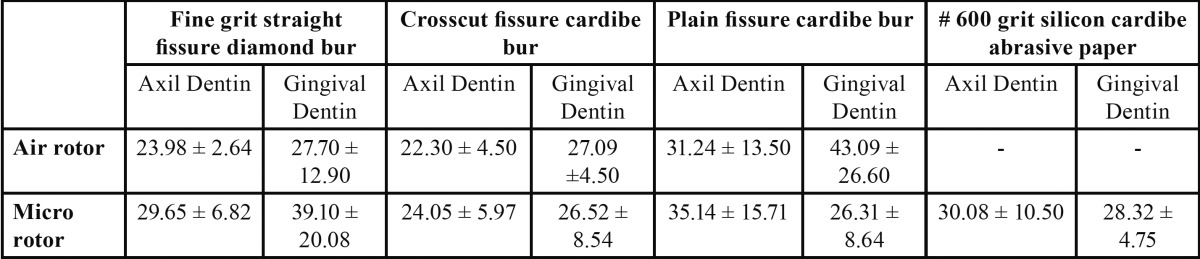
Results of mean gap width (µm) at dentine restoration interface for each group (mean ±SD).

**Figure 2:**
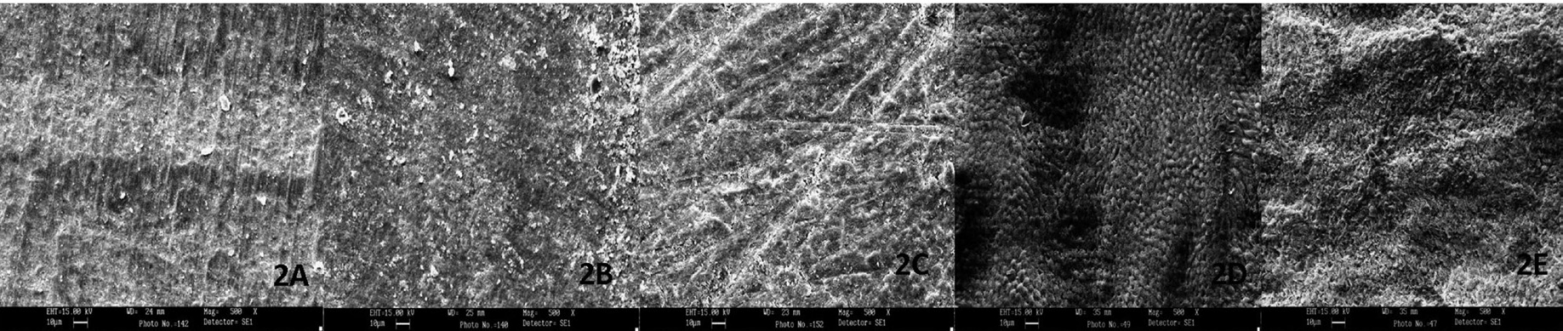
A) SEM view of tooth surface prepared with plain fissure carbide bur in air rotor at 500x, B) SEM view of tooth surface prepared with plain fissure carbide bur in micro motor at 500x, C) SEM view of tooth surface prepared with #600-grit silicon carbide abrasive paper at 500x, D) SEM view of tooth surface prepared with fine grit straight fissure diamond bur in air rotor and conditioned with 38% H3Po4 at 500x, E) SEM view of tooth surface prepared with fine grit straight fissure diamond bur in micro motor and conditioned with 38% H3Po4 at 500x.

SEM observations of surface topography of prepared dentin surfaces are summarized in the photomicrographs (Fig. 1B, 2C). SEM observations revealed that many scratches were left by the abrasive paper or burs and surfaces were completely covered with a smear layer. Plain carbide air rotor (CA) (Fig. 2A), Crosscut carbide air rotor (CCA) (Fig. 1D), Diamond air rotor (DA) (Fig. 1B) were coarser than the other four groups. In other groups, plain carbide micro motor (CM) (Fig. 2B), Crosscut carbide micro motor (CCM) (Fig. 1E), Diamond micro motor (DM) (Fig. 1C) and #600 SiC (Fig. 2C), surfaces were flat & rough with amorphous layer of cutting debris that cover the dentin and obscured the dentinal tubules.

**Figure 3:**
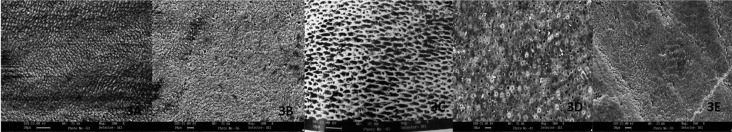
A) SEM view of tooth surface prepared with crosscut fissure carbide bur in air rotor and conditioned with 38% H3Po4 at 500x, B) Scanning electron microscopic view of tooth surface prepared with crosscut fissure carbide bur in micro motor and conditioned with 38% H3Po4 at 500x, C) SEM view of tooth surface prepared with plain fissure carbide bur in air rotor and conditioned with 38% H3Po4 at 500x, D) SEM view of tooth surface prepared with plain fissure carbide bur in micro motor and conditioned with 38% H3Po4 at 500x, E) SEM view of tooth surface prepared with #600-grit silicon carbide abrasive paper and conditioned with 38% H3Po4 at 500x.

SEM of dentin surfaces etched with 38% phosphoric acid revealed complete removal of amorphous smear layer in plain carbide air rotor (CA) (Fig. 3C) followed by plain carbide micro motor (CM) (Fig. 3D). For these groups, intertubular dentin and the peri-tubular dentin orifices were slightly etched, and the edges of the dentinal tubules were clearly observed. In other groups, Crosscut carbide air rotor (CCA) (Fig. 3A), Crosscut carbide micro motor (CCM) (Fig. 3B), Diamond air rotor (DA) (Fig. 2D), Diamond micro motor (DM) (Fig. 2E) , minimum smear layer was removed even after etching. In #600 SiC (Fig. 3E) smear layer was not removed after etching.

## Discussion

A strong, durable and predictable union between restorative resin and the tooth structure is essential for long term success of composite restorations. Polymerization shrinkage is a major concern associated with the use of composite materials. The stresses generated due to polymerization shrinkage may be sufficient to cause de-bonding at resin – tooth interface and leads to gap formation (16). This gap may vary from 1.67 to 5.68% of the total volume of the restoration (17). It has been estimated that a resin to tooth structure bond of 17 Mpa is necessary to offset this effect of polymerization shrinkage and to prevent a micro gap formation between resin and tooth structure (18). The initial polymerization shrinkage of resin composites, the different coefficients of thermal expansion of this material and dental hard tissues and adhesion problems of cervical areas are the essential factors responsible for gap formation and marginal leakage at the resin tooth interface of composite restorations (19).

In this study, the gingival margins of proximal box only cavities were placed in dentin/cementum, below CEJ. As compared to enamel, cementum/dentin is an unstable substrate for bonding. While enamel is almost exclusively an inorganic tissue, dentin is less mineralized and contains more moisture, which can cause variations in adhesion (20). Microleakage at gingival margin with margins in cementum/dentin is one of the weakest links in class II restorations. In cavities where the cervical margin is located on the root dentin apical to cemento-enamel junction, contraction forces may exceed the adhesive strength of the bonding agents to dentin, producing a gap leading to microleakage (21). This is the reason why, in the present study gingival margins of class II cavities were placed below cemento-enamel junction to evaluate the marginal adaptation at cementum/dentin and not enamel. In this in vivo study, interfacial gap was observed at tooth-resin interface in all the specimens regardless of preparation method. Various studies have shown that the bonding performance of various adhesives in vivo studies may not be as good as that shown in vitro studies (21-24). The challenges imposed by the oral environment, such as thermal ﬂuctuations, humidity, chemical challenges and loading stresses constantly threaten the establishment and the longevity of resin-dentin bonds. The contents of dentinal tubules in vital teeth, including dentinal tubules, blood proteins, such as ﬁbrinogen, albumin and immunoglobulin G (IgG), nerve ﬁbers, and unmineralized collagen ﬁbrils may have a significant effect on dentin adhesion. Apart from the outward fluid flow, which is absent in extracted teeth, the surface tension of the dentinal surface may be altered as a result of extraction.

It has been suggested that the hydrostatic pulpal pressure, the dentinal fluid flow and the increased dentinal wetness in vital dentin can affect the intimate interaction of certain dentin adhesives with the dentinal tissue. The presence of fluid inside the dentinal tubules also tends to dilute the dentin conditioner and decrease its potential for demineralization of the intertubular and peritubular dentin, which eventually affects bonding (25). Despite the manufacturers’ instructions that a slight degree of superficial moisture is essential to promote optimal cohesive hybridization, high dentin permeability and the pulpal pressure might have diluted the primer. This would have rendered it less effective resulting in more interfacial gap at composite-tooth interface (21-24).

Various factors contribute to the appearance of gap at resin dentin interface. It has been shown that polymerization shrinkage of resin composites during direct placement causes dimensional strains within the cavity preparation (26). These dimensional strains have been shown to appear as gaps at the tooth-restorative material interface due to the separation of the resin-bonding agent from the underlying dentin, especially along the pulpal wall. The volume of these internal gaps has been reported to be as large as 0.7 µm in Class I restorations (26).

Results of the present in vivo study demonstrated extensive generalized gap formation in all the groups. These results are in contrary to the previous in vitro study [3] reporting a significant gap free attachment of Prime & Bond NT to dentin under moist conditions. Most of the previous studies, which have reported high bond strength, have been performed in vitro on flat dentin surface (27,28). The present study has been performed in vivo conditions in high C-factor cavities which could be a possible explanation for the different results. Moreover the environmental conditions might have also affected the results.

These findings are supported by Hannig & Friedrichs who stated that a completely gap-free adaptation between composite and dentin could not be achieved reproducibly and consistently, in cavities with a high C- factor, either in vivo or in vitro (15).

No significant difference was observed in interfacial gap at axial and gingival dentin. However, more gap was observed in the dentin margin than the enamel margins. This may be because of absence of enamel at gingival margin, poor adaptation of composite at gingival margin due to large occluso-gingival dimension of class II cavity and longer distance of gingival wall from the light source leading to poor adaptation at the gingival margin.

Inadequate adaptation of packable composite resin due to its high viscosity could also be another reason for the gap formation. The bond strength of a stiffer composite is more affected by the cavity configuration than that of a less stiff composite (29). As packable composites are stiffer as compared to micro-hybrid and hybrid composites, the role of C-factor becomes more important.

No effect of bur as well as speed was reported on interfacial adaptation in this study. This can be explained by the fact that etching might have produced similar bonding substrate inspite of different types of smear layer created by different cutting instruments. The given explanation is in accordance with Watson *et al.* who reported no significant differences in subsurface enamel cracking by diamond & carbide bur, however a slight increase in temperature by frictional heat using diamond burs as compared to carbide burs (30). But the above explanation is in contrary to Ogata *et al.* who reported significantly lower bond strength with diamond burs as compared to both smooth and crosscut carbide burs using self-etching systems (27,28). But in this study phosphoric acid was used for etching which removes the smear layer completely. This study is supported by Semeraro *et al.* (31) who observed significantly lower bond strength with regular grit diamond burs in Clearfil SE Bond and SSB 200 while no significant effect of burs was observed in G Bond. Prompt L Pop self etch adhesives depicted lowest bond strength with no significant difference in bond strength with both types of burs and attributed this low bond strength to the complete dissolution of smear layer due to its low pH (0.8). Another reason for no difference in bond strengths may be the presence of “blisters” within the adhesive resin. Blisters may create defects within the adhesive resin during tension and initiate the propagation of cracks within the adhesive. The elimination of blisters should be important for producing good bonding between the resin and dentin. Another study demonstrated that before acid etching, little difference was observed between the surfaces prepared with a steel bur and a diamond bur except the dentinal grooves left by the diamond bur. Both of the surfaces were completely covered with a fairly thick smear layer, and the tubule apertures were not discernible because of this occlusion. Their findings showed a dependence on the bur speeds; there were no differences between the instruments over 25,000 rpm. In this study, the dentin was prepared with a diamond bur at about 380,000 rpm and a carbide steel bur at 40,000 rpm; therefore, little differences was observed on the dentin surfaces between the instruments (8).

Dentin surfaces prepared with diamond burs exhibit an irregular, rough surface with a thick smear layer, traversed by deep grooves. After acid etching, the dentin prepared with the diamond bur found to be covered with a thin smear layer and tubules were barely discernable. In contrast when prepared with the carbide bur the smear layer was thinner, with uniform smooth scratches on the dentin surface. Most of the smear layer was removed after acid etching; the tubule apertures were clearly visible on the prepared dentin. The smear layer produced by the carbide bur was readily removed as compared to the smear layer produced by the diamond bur (8,10).

In most of the studies (10,32) the voltage ranging 12 to 20 has been used so in present study also we preferred 15 KV at which the images were found to be clear and good.

Within limits of our study it was found that different type of burs and their speed may not have any effect on interfacial adaptation of acetone based total etch system at axial and gingival dentin walls. It can be postulated that the stability of the internal seal between composite resin and the acid conditioned dentin in cavities with a high C- factor is still questionable and the optimum dentin surface pre-treatment yet needs to be determined.

Current study did not include the caries affected dentin, silicon carbide paper in air rotor, different type of bonding agents, effect of temperature changes on restoration, effect of longevity on the composite restoration, different type of testing methods and the long term clinical evaluation of the restoration. In order to improve the longevity of adhesive composite resin restorations, further efforts should be directed to an improvement in the adhesive and bonding properties of filling materials placed on the dentin.
